# Comment on: Mesenteric SParIng *versus* extensive mesentereCtomY in primary ileocolic resection for ileocaecal Crohn’s disease (SPICY): study protocol for randomized controlled trial

**DOI:** 10.1093/bjsopen/zrac080

**Published:** 2022-06-03

**Authors:** Deepak Selvakumar, Steven Brown, Laura Hancock

**Affiliations:** 1 Department of General Surgery, Manchester University NHS Foundation Trust, Manchester, UK; 2 Manchester Academic Health Science Centre, University of Manchester, Manchester, UK; 3 Department of General Surgery, Sheffield Teaching Hospitals NHS Foundation Trust, Sheffield, UK

*Dear Editor*

The mesentery in Crohn’s disease has been proposed to be a biological driver of recurrence. Therefore, extent of mesenteric excision may reduce recurrence rates after ileocolic resection. We commend the authors’ aims to investigate this important facet of surgical technique that may influence the efficacy of surgery. Unfortunately, they have not considered the role of the anastomotic technique used^1^. The effect of anastomotic technique is still not clear and there is evidence to suggest that the Kono-S anastomosis in particular may reduce rates of recurrence. There is clearly a clinical need for a high-quality, prospective study on anastomosis to be conducted. This appears to be a missed opportunity to evaluate the effect of anastomotic technique in addition to extent of mesenteric excision on recurrence rates. Mesenteric excision and anastomosis are both key operative steps and conducting a randomized control trial that only evaluates one of these is an inefficient utilisation of resources. An alternative methodology to improve efficiency would be to use a 2 × 2 factorial trial design (close mesenteric resection + Kono-S anastomosis; close mesenteric resection + standard anastomosis; extended mesenteric resection + Kono-S anastomosis; extended mesenteric resection + standard anastomosis). This would provide valuable evidence to determine the most effective surgical procedure to reduce recurrence rates in ileocaecal Crohn’s disease.

*Disclosure*. The authors declare no conflict of interest.
